# Leptin Protein Expression and Promoter Methylation in Ovarian Cancer: A Strong Prognostic Value with Theranostic Promises

**DOI:** 10.3390/ijms222312872

**Published:** 2021-11-28

**Authors:** Mourad Assidi, Fatimah M. Yahya, Maryam H. Al-Zahrani, Razan Elkhatib, Ali Zari, Aisha Elaimi, Jaudah Al-Maghrabi, Ashraf Dallol, Abdelbaset Buhmeida, Muhammad Abu-Elmagd

**Affiliations:** 1 Center of Excellence in Genomic Medicine Research, King Abdulaziz University, Jeddah 21589, Saudi Arabia; mourad.assidi@gmail.com (M.A.); razankhatib@hotmail.com (R.E.); ashrafdallol@gmail.com (A.D.); 2 Department of Medical Laboratory Technology, Faculty of Applied Medical Sciences, King Abdulaziz University, Jeddah 21589, Saudi Arabia; aelaimi@kau.edu.sa; 3 Biochemistry Department, Faculty of Science, King Abdulaziz University, Jeddah 21589, Saudi Arabia; fatyahya23@gmail.com (F.M.Y.); mhsalzahrani@kau.edu.sa (M.H.A.-Z.); 4 Department of Biological Sciences, Faculty of Sciences, King Abdulaziz University, Jeddah 21589, Saudi Arabia; azari@kau.edu.sa; 5 Center of Innovation in Personalized Medicine, King Abdulaziz University, Jeddah 21589, Saudi Arabia; 6 Department of Pathology, Faculty of Medicine, King Abdulaziz University Hospital, Jeddah, 21589, Saudi Arabia; jalmaghrabi@hotmail.com

**Keywords:** ovarian cancer, leptin, methylation, immunohistochemistry, tissue microarray, prognosis, survival

## Abstract

Ovarian cancer (OC) is the deadliest among all gynecological cancers. Epidemiological studies showed that obesity might influence many cancers including OC. One of the key factors that may link obesity and OC is leptin (LEP), known as an adipokine with pleiotropic effects on body homeostasis. This study aims to investigate the expression pattern of LEP, assess the methylation profiles of *LEP* and their associations with clinicopathological features including survival outcomes of OC patients. The protein expression of LEP was evaluated in 208 samples using both tissue microarray and immunohistochemistry techniques. The methylation profiles of *LEP* were measured in 63 formalin-fixed, paraffin-embedded tumor tissues by quantitative polymerase chain reaction using a MethyLight assay. Our results showed a significant association of LEP protein overexpression with several clinicopathological variables, mainly tumor subtype, LVI, age of menarche, tumor size and stage (*p <* 0.04). Kaplan–Meier analysis (using low expression versus high expression as a discriminator) indicated that LEP protein overexpression is a powerful positive prognosticator of both OC recurrence (DFS) and disease-specific survival (DSS) in our OC cohort (log-rank *p* = 0.01 and *p* = 0.002, respectively). This implies that patients with high LEP expression profiles live longer with less recurrence rates. Methylation analysis results demonstrated a clear association between no/low LEP protein expression pattern (38%) and LEP promoter CpG island hypermethylation (43%). Results of this study suggest that LEP is a powerful prognosticator of OC recurrence and DSS. *LEP* expression in OC seems to be regulated by its promoter hypermethylation through gene partial/total silencing. Further multi-institutional studies using larger cohorts are required to demystify the intricate molecular functions of this leptin-driven effects in OC pathophysiology and to accurately assess its theranostic potential and validate its prognostic/predictive power in OC onset, progression towards more effective and personalized management of OC patients.

## 1. Introduction

Ovarian cancer (OC) is the fifth most lethal malignancy among women and the deadliest of all gynecological cancers [1]. In Saudi Arabia, OC is the eighth most common cancer among Saudi females with a four-fold increase in incidence between 1990 and 2016. According to the Saudi cancer registry, the disease affected more than 3% of Saudi women with 409 new cases and 244 deaths in 2018 [2,3,4]. Unfortunately, most patients were diagnosed at advanced stages due to the lack of early signs and symptoms, leading to an increased mortality rate and decreased 5-year survival rate to lower than 45% [5]. Moreover, the poor prognosis of OC is mainly due to the poor understanding of the underlying molecular mechanisms of OC metastasis and progression; thus, the identification of specific biomarkers is urgently needed to improve survival and to develop novel effective therapeutic strategies for OC patients.

Epidemiological studies showed that obesity is a risk factor for many cancers including OC [6]. Every 5 unit increase in body mass index BMI rises the incidence of OC by 6%. Furthermore, obese OC patients have poor survival compared to normal weight OC patients [7,8]. Even though the link between obesity and OC risk and progression has not been fully clarified, many possible mechanisms have been postulated, including hyperinsulinemia, inflammation, fluctuating levels of sex hormones, and adipokines [9,10]. The latter have been identified as cell signaling proteins that are mainly secreted by adipose tissue. The first discovered adipokine was leptin (1994) [11], encoded by the *Ob/LEP* gene located on ch7q32.1, and binds to its receptors (*Ob-R*/*LEPR*) that are found on several tissues, mainly the brain and hypothalamus [12]. LEP, known as the obesity hormone, is also secreted by the ovary, placenta, bone marrow, and mammary gland [13,14,15,16]. *LEP* is regulated by food intake, hormones, metabolites, and cytokines, and its secretion correlates with fat mass and hormones like estrogen, progesterone, and insulin levels [13,17]. 

*LEP* importance resides in maintaining the immunometabolism balance between weight and energy. This occurs by signaling satiety to the hypothalamus subsequently reducing dietary intake and fat storage while modulating energy expenditure and carbohydrate metabolism, preventing further weight gain. Noteworthy, most obese individuals are not leptin deficient but rather leptin “resistant”. Consequently, they have elevated levels of circulating leptin leading the hyperleptinemia [18,19]. Hyperleptinemia has been linked to hypertension, insulin resistance, and the development of cardiovascular and chronic kidney disease [20,21,22,23,24,25,26]. Furthermore, this adipokine (*LEP)* has an established role in angiogenesis, ovulation, and fertilization [27,28,29]. Mutations in the coding or regulatory regions can affect the expression of LEP. For instance, several studies described in detail the polymorphism *LEP* (G2548A) within the 5′ promoter region. This mutation has been associated with variations in BMI, the concentrations of LEP in the plasma [30,31,32,33], and high-density lipoprotein cholesterol (HDL-C) levels [34]. 

The involvement of the LEP in many diseases, as previously mentioned, has also been reported in various types of cancer. Previous data reported overexpression of LEP protein in renal carcinoma [35] and breast carcinoma [36]. Recently, the LEP protein was shown to promote cancer progression and metastasis by regulating the epithelial–mesenchymal transition (EMT), cell adhesion to the extracellular matrix (ECM), and proteolysis of the ECM components [37,38,39,40]. Increasing evidence showed the link between *LEP* dysregulation and OC development, however, the exact mechanism and prognostic value of leptin remain unclear. In this study, we assessed the expression levels of LEP in OC tissues and its correlation with the methylation status of the *LEP* promoter region. Finally, we assessed its prognostic value in our cohort. 

## 2. Results

### 2.1. Patients Overview

As shown in Table 1, more than 56% of the patients diagnosed with OC were younger than 50 years of age. Furthermore, most of the cohort we studied were premenopausal (57%), and/or obese (BMI > 26) women. Surprisingly, more than half of the OC patients were diagnosed at advanced stages (III and IV) and high grades, where the sizes of the eradicated tumors were bigger than 10 cm^3^. Also, nearly half of the cohort was diagnosed with the serous histological subtype.

### 2.2. LEP Protein Expression Pattern

**Figure 1:**
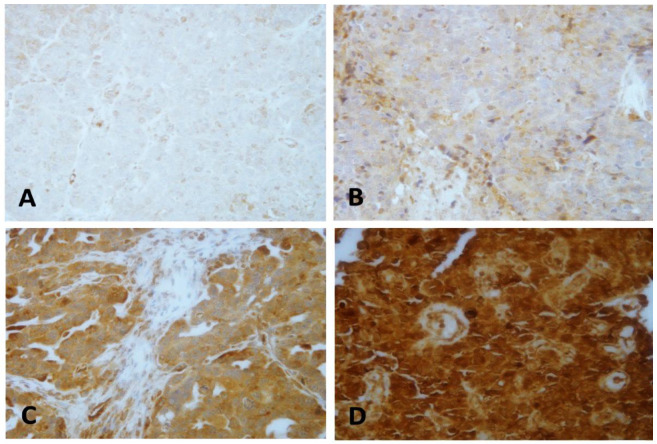
Various levels of cytoplasmic leptin protein expression patterns: (**A**) no (0) expression pattern, (**B**) weak (1+) cytoplasmic expression pattern, (**C**) moderate (2+) cytoplasmic expression pattern, (**D**) strong (3+) cytoplasmic expression pattern of leptin protein. Magnification ×40.

Immunohistochemical (IHC) analysis was performed in tissue microarray (TMA) slides to assess the expression pattern of LEP protein. Analyzed OC tissue samples showed diffuse cytoplasmic localization of LEP expression. According to the suggested cut-off point of evaluation (low expression vs. high expression), 24% of our OC tissue samples showed low (0, 1+) cytoplasmic expression of LEP protein as compared to 76% of high cytoplasmic expression pattern (Figure 1).

### 2.3. LEP Protein Expression Pattern and Clinicopathological Features

The association of LEP expression pattern with the OC patients’ clinicopathological features was assessed using (low expression versus high expression) as the best discriminator (Table 1). LEP protein expression showed significant correlations with the tumor subtype (*p =* 0.001), LVI (*p =* 0.001), age of menarche (*p =* 0.01), tumor size (*p =* 0.01) and stage (*p =* 0.04). 

### 2.4. LEP Protein Expression and Survival Outcomes

**Figure 2:**
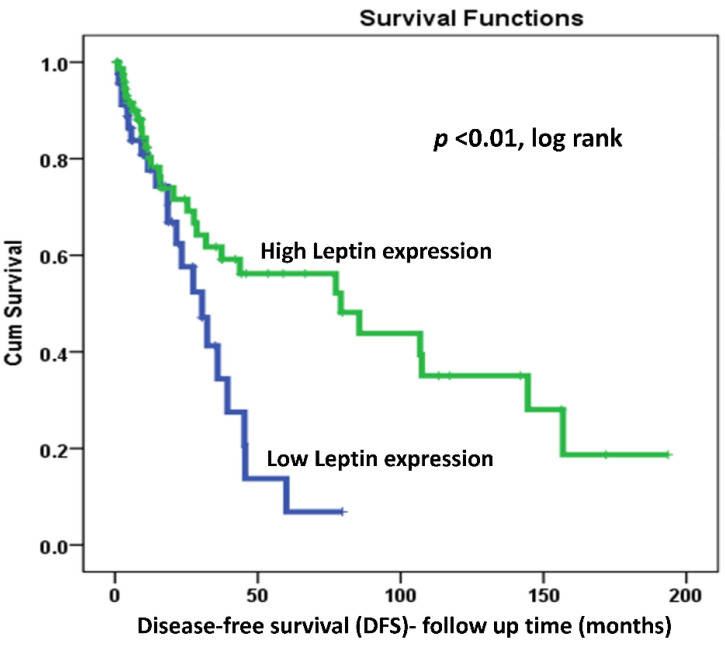
Kaplan–Meier analysis of the association between LEP protein expression pattern and Disease-Free-survival (DFS) in OC patients using (low vs. high expression as a discriminator (*p* = 0.01, log-rank).

**Figure 3:**
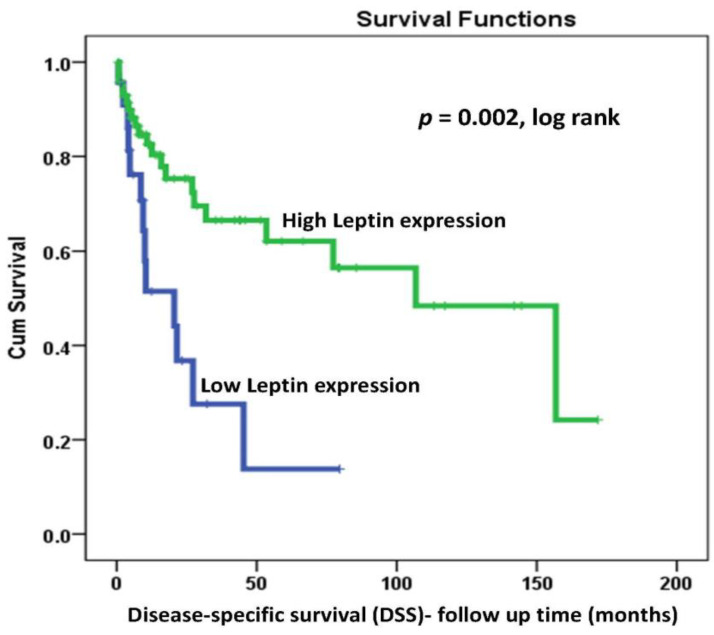
Kaplan–Meier analysis of the association between LEP protein expression pattern and Disease-Specific-Survival (DSS) in OC patients using (low versus high expression as a discriminator (*p* = 0.002, log-rank).

Disease-Free Survival (DFS) and Disease-Specific Survival (DSS) data using Kaplan–Meier analysis and (low expression versus high expression) as a discriminator indicated that both DFS and DSS were significantly associated with LEP protein expression in OC patients (log-rank *p =* 0.01 and *p* = 0.002, respectively). In addition, LEP protein overexpression was a powerful positive prognosticator of both OC recurrence and DSS in our cohort. Interestingly, more than half of our patients’ cohort with high LEP expression were alive (Table 1). In fact, at 60 months follow-up time, only 43% of patients with LEP overexpression relapsed compared to 90% of their counterparts with lower LEP expression (Figure 2). Moreover, and for the same 60 months follow-up time, 62% of patients with LEP overexpression were alive compared to only 18% of their counterparts with lower LEP expression (Figure 3).

### 2.5. Association of LEP Methylation Profiles with Clinicopathological Features and Survival Outcomes

To further investigate the LEP expression pathways in OC, we analyzed the methylation profiles of the *LEP* gene promoter in 63 OC patients. The inclusion criteria were based on the availability of the tissue samples and patients’ medical record data. Our results demonstrated that *LEP* promoter was hypermethylated in 43% (27/63) of our OC patient cohort. Interestingly, our data showed that LEP methylation profiles were mainly correlated with histological subtype (*p =* 0.001), tumor site (*p =* 0.04), menopausal status (*p =* 0.09), and patients’ age (*p =* 0.06). No association was noted between *LEP* methylation in OC patients and BMI and tumor stage (*p* > 0.05) (Table 2). 

For the survival outcomes, Kaplan–Meier survival analysis revealed that there was no association between either DSS or DFS with *LEP* methylation profiles (*p* > 0.05, log-rank).

## 3. Discussion

Ovarian cancer is a disease marked by the absence of early specific signs and silent clinical symptoms making its diagnosis at an early stage challenging, thus leading to higher mortality rates, being the highest deadly cancer among all gynecological tumors [5,41,42,43]. In this context, lipid metabolism and obesity were reported as the main risk factors for several chronic diseases such as chronic inflammation, cardiovascular diseases, diabetes, bone degenerative diseases and cancer, which has urged scientists to investigate the underlying biological and molecular links [38,39,44,45,46,47,48]. Furthermore, several studies have linked leptin expression with several upstream (pathways inducing LEP expression) and/or downstream (pathways induced by LEP) inflammatory-like pathways that are involved in obesity-associated diseases mentioned above, mainly cancer [48,49]. In fact, a strong association between obesity and many cancers’ susceptibility including OC was reported [50,51], where obese women have a two-fold increased risk of premenopausal OC [52]. Remarkably, women with a higher BMI reported having symptoms for a longer time before being diagnosed with OC [53]. In this context, leptin is a key adipocyte-derived factor involved in energy balance, appetite regulation, nutrient intake, energy balance, metabolic homeostasis, adipose tissue expansion, obesogenic pathways, and central nervous insensibility to leptin (loss of responsiveness, “type 2 obesity”) [54,55]. Moreover, in vitro studies reported that high levels of LEP stimulate proliferation, cell migration, invasion and inhibit apoptosis of OC cells [56,57,58]. In this direction, it is reported that the involvement of *LEP* in the process of tumorigenesis and metastasis is mediated through regulating the EMT, cell adhesion and proteolysis of the ECM components [37]. The involvement of LEP in lifestyle-diseases risk factors (through obesity pathophysiological pathways) also supports the possible impact of the environment and epigenetic pathways in LEP function and/or regulation. 

These findings suggested possible links between *LEP* expression and OC, however, the exact mechanism of this gene and its potential prognostic value remain unclear. Furthermore, several studies have investigated the protein expression pattern of LEP in tissues of many cancer types such as breast [59,60] and colorectal cancer [61], but only one study had assessed the clinical value of LEP and its associated receptor (Ob-R) in OC patients from the Middle-East region [62]. In the current study, we evaluated the protein expression of LEP in OC tissues, its correlation with the *LEP* promoter methylation status, and its potential prognostic value. Our IHC results showed a cytoplasmic (Figure 1) expression with significant association with several clinicopathological features including tumor subtype, lympho-vascular invasion (LVI), age of menarche, tumor size and stage (*p* < 0.04) (Table 1). Compared to our results, Uddin et al. did not report any association of LEP expression with age, histology type, tumor grade, stage, and BMI. In line with our findings, the authors did not establish any correlations between LEP expression and age, tumor grade, and BMI (*p* > 0.05) [62]. Similar studies found no association between LEP levels with clinicopathological parameters of OC patients including age, grade, histological subtype, and BMI [63,64,65,66,67,68]. Other reports showed an inverse correlation between LEP levels and advanced stages of OC [64,69]. Likewise, some studies noted a positive correlation between LEP levels and BMI in healthy women but not in early and advanced OC patients. Despite some reports linking LEP, obesity and OC, the molecular mechanism behind it is still poorly understood [38,39,40].

Interestingly, Kaplan–Meier survival analysis of our cohort showed a strong prognostic power of LEP protein expression. Indeed, patients with LEP overexpression had a strong trend toward better prognosis marked by lower recurrence (DFS; log-rank *p =* 0.01; Figure 2) and reduced disease-specific deaths (DSS, log-rank *p* = 0.002; Figure 3). In fact, at 60 months follow-up time, only 43% of patients with LEP overexpression relapsed compared to 90% of their counterparts with lower LEP expression (Figure 2). Moreover, and for the same 60 months follow-up time, 62% of patients with LEP overexpression were alive compared to only 18% of their counterparts with lower LEP expression (Figure 3). These survival results support an “antitumoral-like” role of leptin that could be attributed to a more effective response to cancer therapies in obese patients, named also the “leptin paradox” in cancer as reviewed recently by Sánchez-Margalet’s group [70]. Furthermore, our results showed a noticeably higher LEP expression at poorly differentiated advanced stages. However, the Kaplan–Maier survival analysis, which is a univariate analysis that evaluated only the correlation of LEP expression versus either disease recurrence or death, showed lower recurrence and better survival rates. In fact, it seems that OC patients with higher LEP expression have an advantage compared to their counterparts since they behave better and survive longer irrespective of the other clinicopathological features. Possible LEP involvement in anti-tumor activity and/or in the enhancement of treatment effectiveness discussed above could justify these results. While it has been reported that OC patients who were suffering from obesity (higher LEP expression/serum levels) had worse overall survival and progression-free survival [52,71], other studies reported an inverse relationship between LEP levels and the survival rates of OC patients. To elaborate further, higher levels of LEP were associated with shorter DFS and poorer DSS [56,67,72,73]. Leptin receptor (LEPR/ObR) overexpression was also shown to be significantly associated with OC poor progression-free survival [62].

To investigate further possible gene expression pathways and roles of LEP in OC pathophysiology and prognosis, we have investigated the *LEP* promoter methylation in our cohort. Certainly, mutations in *LEP*’s promoter region had significant correlations with circulating LEP levels and BMI [34,74,75]. Additionally, DNA methylation levels of the *LEP* gene were shown to be correlated with BMI [76]. To the best of our knowledge, this is the first study to evaluate the methylation profiles of the promoter region of the *LEP* gene in OC patients. Our data revealed that the *LEP* promoter was hypermethylated in approximately 43% (27/63) of OC patients. Interestingly, our data showed that *LEP* methylation profiles were correlated mainly with histological subtype, menopausal status, and patients’ age (*p >* 0.05), but no significant associations were found with BMI and tumor stage (*p* > 0.05) (Table 2).

Despite the absence of significant associations between the *LEP* promoter methylation status and patients’ survival outcomes (DFS and DSS) (data not shown), our results demonstrated a clear association between *LEP* promoter hypermethylation profiles and a no/low LEP protein expression pattern. In reality, 43% of *LEP* promoter hypermethylation in our cohort is approximately matching with the proportion of OC patients with no/low LEP protein expression (38%) of OC patients (Table 3). 

**Figure 4:**
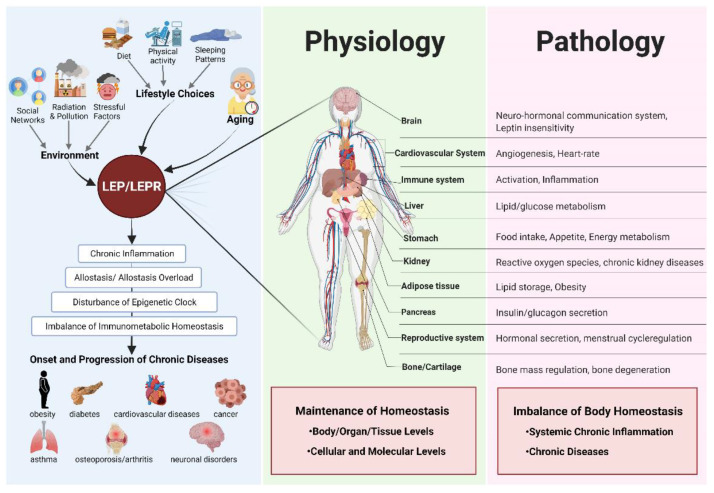
An overview of the key roles of “leptin switch” in homeostasis disturbance and pathophysiology of complex diseases.

Therefore, these concordances between IHC and methylation analysis results confirm our hypothesis that *LEP* promoter hypermethylation might be the main factor in LEP protein expression silencing. This no/low expression could hence be due to the established epigenetic process known as promoter hypermethylation through gene partial/total silencing [77,78]. Our results showed clearly that *LEP* methylation profiles affect the regulation and expression of the LEP protein in OC. The regulation of several cancer biomarkers by these epigenetic events (mainly promoter hypermethylation) were documented in several studies [77,78]. Our results confirm the previous reports highlighting possible effects of environmental conditions and lifestyle choices as key stressful factors that progressively induce tissue-specific adaptation processes (allostasis processes) to maintain homeostasis. This occurs through the activation of several signaling and metabolic pathways, as well as through the modulation of the epigenetic/epigenomic clock [79,80,81,82,83,84]. These cumulative “stress” factors will over time exert an immunometabolic overload and induce homeostasis disturbances that favor the onset and/or the progression of chronic diseases (Figure 4).

Our results showed a strong prognostic value of LEP that could be of high clinical utility towards a better stratification and management of OC patients. Furthermore, we assume that the lack of significant correlations between BMI and LEP expression in our findings could be attributed to the small size and the heterogeneity of our cohort. In other studies, however, the leptin epigenetic profile was associated with obesity and increased risk of chronic diseases, mainly cancer in human and/or functional models [85,86,87]. These discrepancies could also be due to the fact that BMI is not a very accurate parameter to predict obesity status [70]. Other factors such as leptin circulating levels, the gene expression pattern of both *LEP* and its receptor LEPR, the epigenetic events (DNA CpG islands methylation events as shown in this study) affecting the *LEP* gene, the LEPR (Ob-R) polymorphism (including the long active isoform Ob-Rb, the short LEPR isoforms (Ob-Ra, Ob-Rc, Ob-Rd), and the soluble LEPR isoform (Ob-Re)) should be considered to better understand the downstream LEP signal transduction pathways to define more accurately both the LEP prognostic and predictive value, and to refine the cohort of cancer patients that will have better outcomes [70,84,88]. The involvement of several genes and multiple molecular/signalling oncogenic pathways (including growth factors, Notch, and pro-inflammatory signalling) events along with *LEP* gene promoter methylation, protein expression, and the obesity-related metabolic events highlight the magnitude and complexity of LEP molecular functions in both health or disease as discussed earlier (Figure 4) [49,83]. Further multi-institutional studies using larger cohorts are required to demystify the intricate molecular functions of this “leptin switch” from physiology to pathology, tailor leptin-based theranostic strategies, assess more accurately its prognostic/predictive power in OC onset, progression, as well as ensure more personalized management of OC patients. This study is a part of an Oncoscreen project at King Abdulaziz University which aims at identifying cancer inducers and discovering cancer biomarkers, an approach towards more accurate cancer prevention, early detection, prognosis and prediction.

## 4. Materials and Methods

### 4.1. Patients and Samples

This retrospective study included 208 OC patients’ formalin-fixed paraffin-embedded (FFPE) tissue collected in the Departments of Pathology and Gynecology, King Abdulaziz University Hospital (KAUH) between 1995 and 2014. It has been identified primarily by the Tumor Node Metastasis (TNM) classification system using histopathological features. The tissue samples and the associated clinical data were collected after all the necessary ethical approvals were obtained in compliance with the guidelines of the King Abdulaziz University Hospital Ethical Committee (Ref. number: KAU-189-14). The patients’ clinicopathological characteristics (age, menopausal status, stage, grade, and status of the lymph node), follow-up data, and survival are summarized in Table 1.

### 4.2. Tissue Microarray

TMA was generated in-house from the archived FFPE tissue blocks. After sections preparation, the slides were stained with hematoxylin and eosin (H&E). Five TMA recipient paraffin blocks used in this study were produced. The TMA contained duplicate specimens of the OC patient and duplicate specimens of the placenta as control tissue. 

### 4.3. Immunohistochemistry

Immunohistochemical staining of 5 TMA slides derived from 208 patients diagnosed with OC in KAUH, Jeddah was performed using an automated titration run staining system (Benchmark XT, Ventana Medical System, Inc., Tucson, AZ, USA), which allows manual addition of antibodies. The rabbit polyclonal antibody against LEP (sc-842, Santa Cruz, CA, USA) was used at the dilution of 1:50.

#### 4.3.1. Evaluation of LEP Protein Expression Pattern

LEP protein staining was evaluated using a standard x40 magnification light microscope, blinded by tumor grade, point, or clinical outcome details. A four category (0, 1+, 2+, 3+) scoring system was adopted for cell cytoplasmic staining: (0) means no expression, no detectable stain in <10% of the membrane; (1+) mild yet detectable discontinuous stain present in 10–39% of the membranes; (2+) moderate, clearly positive discontinuous stain present in 40–90% of the membranes; and (3+) strong continuous membrane stain. With both the staining intensity and the fraction of positively stained cells considered using the following formula, the staining index of the membranous expression pattern was calculated based on the following equation:

I = 0 ∗ f0 + 1 ∗ f1 + 2 ∗ f2 + 3 ∗ f3

where I: is the staining index; f0–f3 are the fractions of the cells showing a defined level of staining intensity (from 0 to 3). Theoretically, the index scores could vary between 0 and 3 [89,90]. The reproducibility of the evaluation of Leptin staining indices was tested by employing intra-observer reproducibility.

#### 4.3.2. Statistical Analysis 

Statistical analyses were performed using the SPSS^®^ (IMB, Armonk, NY, USA) software packages (PASW Statistics for Windows, version 19). Frequency tables were analyzed using the Chi-square test, with Fischer’s exact test to assess the significance of the correlation between the categorical variables. Univariate survival analysis for the outcome measure (DSS, DFS) was based on the Kaplan–Meier method, with log-rank (Mantel–Cox) comparison test. In all tests, the values *p* < 0.05 were considered as statistically significant.

### 4.4. Methylation Profiling of 63 FFPE Tissue Samples

#### 4.4.1. DNA Extraction

Xylene (Sigma-Aldrich, Steinheim am Albuch, Germany) was used for paraffin dissolution to extract DNA from FFPE rolls followed by washing with 100% ethanol (Sigma-Aldrich, Steinheim am Albuch, Germany). After the deparaffinization step, the DNA was extracted from tissues using the QIAamp DNA FFPE Tissue Kit (Qiagen) as directed by the manufacturer. The concentrations of all the DNA samples were measured using the Thermo Scientific NanoDrop 2000 Spectrophotometer.

#### 4.4.2. Methylation Analysis of the Candidate Genes

As detailed in the manufacturer’s manual and stated elsewhere [91], bisulfite treatment of the *LEP* promoter region was performed using (Qiagen EpiTect ^®^ Bisulfite Conversion). Briefly, NaH_2_SO_4_ was incubated with 0.5 μg of the extracted DNA. The unmethylated cytosine residues were converted into uracil residues, while the methylated ones remained as it is (cytosine). For MethyLight assay, EpiTect ^®^ MethyLight PCR Kit (cat. No.59496 QIAGEN Inc., Germantown, MD, USA) and EpiTect ^®^ Control DNA and Control DNA Package (cat. No.59695 QIAGEN Inc., Germantown, MD, USA) were used. Specially designed primers and fluorescent dual-labeled TaqMan probe were produced (Probe 6FAM- TTAAGGGTGCGCGCGTGGTTT-BHQ1, Forward primer 5’-AGGGATATTAAGGATTTTTC-3’, Reverse primer 5’-ACCTCGAAAAAAAACCTCGAC-3’) were used for DNA amplification. After that, we proceeded with a qRT-PCR machine using StepOnePlus ™ -Real-time PCR system (Applied biosystems, serial No. 272-000416) by placing the qPCR master mix with the DNA samples. 

#### 4.4.3. qPCR Statistical Analysis

Statistical analyses were performed using the SPSS^®^ (IMB, Armonk, NY, USA) software packages (PASW Statistics for Windows, version 19). To identify the statistically significant correlation between hypermethylation events and clinicopathological features of our cohort, we used ANOVA followed by Fisher’s exact test. Furthermore, DSS and DFS were calculated by univariate Kaplan–Meier analysis, and equality of the survival functions was determined by log-rank (Mantel-Cox) test, withal, *p*-values < 0.05 were considered statistically significant.

## Figures and Tables

**Table 1:** Baseline characteristics (clinicopathological features) of our OC patient cohort, and their correlation with LEP protein expression.

Features	Total Number of Patients N (%)	Number of LEP Expression Cases (%)		*p*-Value
		Low Expression (0, 1+)	High Expression (2+, 3+)	
**Age**				
<50	117 (56%)	22%	78%	0.77
>50	90 (43.5%)	20%	80%	
Missing	1 (0.5%)			
**Tumor Site**				
Right	36 (17%)	20%	80%	0.52
Left	24 (12%)	37%	63%	
Bilateral	146 (70%)	20%	80%	
Missing	2 (1%)			
**Tumor Size**				
1–5 cm	44 (21%)	14%	86%	**0.01**
6–10 cm	50 (24%)	4%	96%	
>10 cm	98 (47%)	33%	67%	
Missing	16 (8%)			
**Tumor Subtype**				
Serous	96 (46%)	0%	100%	**0.001**
Mucinous	39 (19%)	17%	83%	
Other	63 (30%)	43%	57%	
Missing	10 (5%)			
**Tumor Grade**				
Low grade	29 (14%)	8%	92%	0.24
Intermediate	35 (17%)	32%	69%	
High grade	107 (51%)	14%	86%	
Missing	37 (18%)			
**LVI Status**				
Positive	67 (32%)	39%	61%	**0.001**
Negative	106 (51%)	7%	93%	
Missing	35 (17%)			
**BMI**				
<23	19 (9%)	0%	100%	0.20
23–26	52 (25%)	41%	59%	
>26	88 (42%)	23%	77%	
Missing	49 (24%)			
**Parity**				
Parous	98 (47%)	31%	69%	0.07
Nulliparous	64 (31%)	49%	51%	
Missing	46 (22%)			
**Age of Menarche**				
<13	58 (28%)	57%	43%	**0.01**
>13	99 (48%)	20%	80%	
Missing	49 (24%)			
**Menopausal Status**				
Premenopausal	118 (57%)	26%	74%	0.29
Postmenopausal	88 (42%)	17%	83%	
Missing	2 (1%)			
**Tumor Stage**				
Stage I	59 (28%)	28%	72%	**0.04**
Stage II	17 (8%)	50%	50%	
Stage III	85 (41%)	9%	91%	
Stage IV	30 (15%)	29%	71%	
Missing	17 (8%)			

**Table 2:** Correlation between LEP methylation profile and clinicopathological features of OC patients. “**0**”: unmethylated LEP promoter; “**1**”: hypermethylated LEP promoter.

Feature	Number of Cases (%)	*LEP* Methylation Profile		*p*-Value
		“0” (%)	“1” (%)	
**Age**				
<50	40 (64%)	19 (79%)	21 (55%)	**0.06**
>50	22 (35%)	5 (21%)	17 (45%)	
Missing	1 (2%)			
**Tumor Site**				
Right	11 (18%)	4 (17%)	7 (18%)	**0.04**
Left	11 (18%)	8 (33%)	3 (8%)	
bilateral	40 (64%)	12 (50%)	28 (74%)	
Missing	1 (2%)			
**Tumor Size**				
1–5 cm	18 (29%)	9 (39%)	9 (24%)	0.39
6–10 cm	16 (25%)	6 (26%)	10 (26%)	
>10 cm	27 (43%)	8 (35%)	19 (50%)	
Missing	2 (3%)			
**Tumor Subtype**				
Serous	30 (48%)	12 (50%)	18 (47%)	**0.001**
Mucinous	17 (27%)	1 (4%)	16 (42%)	
Other	14 (22%)	11 (46%)	3 (8%)	
Unknown	1 (2%)	0 (0%)	1 (3%)	
Missing	1 (2%)			
**Tumor Grade**				
Low grade	8 (13%)	4 (20%)	4 (13%)	0.80
Intermediate	15 (24%)	5 (24%)	10 (32%)	
High grade	19 (30%)	7 (33%)	12 (39%)	
Missing	21 (11%)			
**LVI Status**				
Positive	28 (44%)	7 (54%)	21 (68%)	0.38
Negative	16 (25%)	6 (46%)	10 (32%)	
Missing	19 (30%)			
**BMI**				
<23	2 (3%)	0 (0%)	2 (10%)	0.11
23–26	12 (19%)	8 (47%)	4 (19%)	
>26	24 (38%)	9 (53%)	15 (71%)	
Missing	25 (40%)			
**Parity**				
Parous	29 (46%)	10 (63%)	19 (73%)	0.47
Nulliparous	13 (20%)	6 (38%)	7 (27%)	
Missing	21 (33%)			
**Menopausal Status**				
Premenopausal	41 (65%)	19 (79%)	22 (58%)	0.09
Postmenopausal	21 (33%)	5 (21%)	16 (42%)	
Missing	1 (2%)			
**Tumor Stage**				
Stage I	16 (25%)	8 (38%)	8 (23%)	0.64
Stage II	4 (6%)	1 (5%)	3 (9%)	
Stage III	31 (49%)	10 (48%)	21 (60%)	
Stage IV	5 (8%)	2 (10%)	3 (9%)	
Missing	7 (11%)			

**Table 3:** Concordance between LEP protein expression profiles and their promoter methylation patterns in Saudi OC patients.

LEP Protein Expression Patterns		*LEP* Promoter Methylation Status	
No/Low expression	38%	Hypermethylation	43%
High expression	62%	Unmethylated	57%
